# Adverse Reactions from Topical Ophthalmic Anesthetic Abuse

**DOI:** 10.18502/jovr.v17i4.12297

**Published:** 2022-11-29

**Authors:** Ali Sharifi, Naser Naisiri, Majid Shams, Meraj Sharifi, Hamid Sharifi

**Affiliations:** ^1^Department of Ophthalmology, Shafa Hospital, Afzalipour School of Medicine, Kerman University of Medical Sciences, Kerman, Iran; ^2^HIV/STI Surveillance Research Center, and WHO Collaborating Center for HIV Surveillance, Institute for Futures Studies in Health, Kerman University of Medical Sciences, Kerman, Iran; ^3^Afzalipour School of Medicine, Kerman University of Medical Sciences, Kerman, Iran; ^5^https://orcid.org/0000-0003-0713-088X

**Keywords:** Adverse Drug Reactions (ADR), Case Series, Ophthalmic Anesthetic, Tetracaine

## Abstract

**Purpose:**

To assess the adverse drug reactions (ADR) of tetracaine among patients referred to an eye emergency department in the southeast of Iran.

**Methods:**

In this case series study, we assessed 31 eyes of 24 patients who were referred due to adverse effects of ocular anesthetics during2017–2020. We collected the data, including age, sex, job, how the medicine was obtained, symptoms, examination results, and ADR.

**Results:**

Of 24 patients, 22 (91.7%) were male. The mean (standard deviation) age of the patients was 32.6 (1.9) years. Twenty-two patients obtained the medicines without a prescription and a general practitioner prescribed the medicine to two patients. In the first interview, the most common symptoms were: photophobia, reduced vision, ocular pain, and redness. The main signs of persistent epithelial defect, patchy or diffuse corneal stromal infiltration, ring infiltration, and Descemet's folds were noticed in the examinations. Finally, 51.6% (*n* =16) of the eyes had decreased vision, 45.2% (*n* =14) had corneal opacity, 16.1% (*n* = 5) had elevated intraocular pressure that needed long-term anti-glaucoma therapy, and 6.5% (*n* = 2) had corneal pannus. Corneal perforation and phthisis bulbi were the final results in one eye.

**Conclusion:**

ADR related to the use of ophthalmic topical anesthetics could cause sight-threatening severe morbidities. It seems that some general practitioners are not careful regarding the prescription of these kinds of medicine. Moreover, the over-the-counter availability of tetracaine eye drops should be managed.

##  INTRODUCTION

Ophthalmic anesthetics are commonly used in daily ophthalmologic practices for diagnostic purposes or therapeutic procedures (such as removing ocular surface foreign bodies and ocular surgeries). It was indicated that the uncontrolled usage of these drugs could cause severe, vision-threatening adverse effects.^[1,2]^ Topical anesthetics can cause both dose- and time-dependent cytotoxicity to human corneal stromal cells, resulting in cell growth inhibition, retardation of cell proliferation, morphological abnormalities, and decreased viability of these cells. Corneal stromal cells treated with tetracaine showed some features associated with apoptosis, such as the increased permeability of plasma membrane and fragmentation of DNA.^[3]^ The symptoms related to ophthalmic anesthetics may be misdiagnosed, so they must be included in the differential diagnosis of chronic infectious keratitis like acanthamoeba keratitis. ^[2]^

In developed countries, these drugs are classed as prescription-only medications (POM) rather than over-the-counter (OTC) medications. However, these medicines can be obtained liberally with no prescription in some parts of the world.^[4,5]^ As there is no promising treatment plan to relieve ocular symptoms due to corneal abrasions and ultraviolet keratopathy, such as pain, the people under such conditions, one way or other, seek anesthetics as a means to achieve symptom relief.

Self-treatment is frequent in developing countries such as Iran where patients could purchase this type of medicine easily. The prevalence of self-treatment in Iran varies between 35.4% and 83%.^[6,7]^ Usually, self-treatment is higher in certain underprivileged populations. Self-treatment might be the first choice for this population because of a lack of knowledge about eye health and the adverse effects of these medicines. Moreover, low health insurance coverage and limited access to healthcare facilities could be other factors that motivate them to use these medicines without prescription. These conditions could increase the burden of the adverse effects from using these kinds of medicines.^[6]^

Although the implications of the ophthalmic anesthetics, especially tetracaine, have been documented in literature, little evidence is available on the complications that may exist in developing countries such as Iran,^[6]^ where people could obtain these kinds of medicines easily. Therefore, it is necessary to study the adverse events and the respective harm caused when using these medicines to increase public knowledge. This study evaluated a case series of patients with topical ocular anesthetic abuse and adverse drug effects who were referred to the ophthalmology department of Shafa Medical Center, Kerman city, southeast of Iran.

##  METHODS

In the present study, 24 patients (31 eyes) with adverse drug reactions (ADR) due to topical anesthetic eye drops were evaluated. All patients were residents in Kerman province, located in the southeast of Iran, and were referred between March 2017 and March 2020. The consent of the patients was obtained to publish the data. The study's protocol was reviewed and approved by the Ethics Committee of Kerman University of Medical Sciences (IR.KMU.AH.REC.1393.401).

A cornea subspecialist (AS) performed eye examinations, managed the patients, and supervised data collection. The collected data included patients' age, sex, job, place of obtaining medication, how the drug was obtained, who introduced the drug, previous history of drug usage, the main complaints, symptoms, signs, examination results, duration of hospitalization, cause of the initial injury, duration of drug usage, and vision. In the current research, we had a uniform protocol for the treatment of all the included patients. The offending agent was discontinued, and a topical antibiotic (ciprofloxacin or chloramphenicol or sulfacetamide10%), a topical steroid (prednisolone acetate or fluorometholone), and frequent preservative-free artificial tears were initiated. The drugs were tapered based on the response to therapy. The results of the first examination (at the time of admission), examination at the time of discharge, and the final examination after treatment (6–12 months after the reaction) were evaluated. Data was described using descriptive statistics for categorical variables (proportion) and continuous variables (mean ± standard deviation (SD)) using Stata 14.2.

##  RESULTS

All patients disclosed the abuse of anesthetic drops at admission; therefore, other pathologies were ruled out in the differential diagnosis. The mean ± SD (range) of the patients' age was 32.6 ± 1.9 years (18 to 57). Most of the included patients (91.7%; *n* = 22) were male. In addition, 58.3% (*n* = 14) of the patients stated that their job was welding. As an underlying problem for the use of tetracaine in 87.5% (*n* = 21) of the subjects was workplace. All patients obtained tetracaine eye drops from a pharmacy, and 91.7% (*n* = 22) obtained this drug with no prescription. Of note, 50% (*n* = 12) of the patients had a history of previous use of tetracaine eye drops. The underlying conditions leading to topical anesthetic misuse were ultraviolet keratitis (in 45.3% of the cases; *n* = 14), ocular foreign bodies (in 22.6% of the cases; *n* = 7), and dry eye symptoms (in 9.6% of the cases; *n* = 3), respectively. Main clinical signs after using the tetracaine eye drops at the first examination were as follows: persistent epithelial defect (PED) (90.3%; *n* = 28), diffuse or patchy corneal infiltration (51.6%; *n* = 16), corneal ring infiltration (32.3%; *n* = 10), and Descemet's folding (32.3%; *n* = 10) [Table 1].

Most of the study patients (58.3%; *n* = 14) had used more than one bottle of tetracaine. It is noteworthy that (37.5%; *n* = 9) of the patients had a bilateral injury. In total, (83.3%; *n* = 20) of the subjects were admitted to be treated, and the others were followed-up as outpatient cases. Duration of their hospitalization was 6.5 ± 0.99 days ranging from zero to 17 days. The mean ± SD time from starting the use of topical tetracaine to the presentation of adverse events was 9.03 ± 1.7 days [Table 2].

All 31 eyes had visual deficit at presentation (VA was hand motion to 0.6) [Table 3]. While the Mean ± SD of Log MAR of vision at the time of hospitalization was calculated as 1.2 ± 0.11 (20/400 Snellen VA), it was 0.8 ± 0.11 (20/125) at the time of discharge and 0.16 ± 0.11 (20/30) at the final examination. The main complaints included pain (93.5%; *n* = 29) and reduced vision (77.4%; *n* = 24). Most of the symptoms in the initial interview were reported as photophobia (96.8%; *n* = 30), reduced vision (96.8%; *n* = 30), and redness and pain (93.5%; *n* = 29). In addition, PED (90.3%, *n* = 29), diffuse or patchy infiltration (51.6%; *n* = 16), and ring infiltration (32.3%; *n* = 10) were the most observed signs in the examinations performed by the ophthalmologist [Table 1 & Figure 2]. A 19-year-old housewife with no history of any underlying disease was admitted with bilateral diffuse corneal haziness, severely reduced vision (hand motion), and corneal epithelial defect after instillation of only two drops of tetracaine. The main reason for referring the patient to the doctor was a severely dry eye. She was brought to a local medical center with dry eye symptoms, including redness, eye burning, and foreign body sensation. After she was admitted at our center, sulfacetamide 10% and prednisolone acetate 1% eye drops was prescribed to her; the ocular injuries, including diffuse and full thickness corneal haziness and central corneal epithelial defect were improved within three days, and then the patient was discharged. Accordingly, in this patient, the corneas became clear within three days of treatment, which improved vision.

**Table 1:** Demographic characteristics, reason of drug misuse (initial injury), and clinical findings at the first examination and patient's main complaints and symptoms of the patients who were referred to eye emergency department for adverse drug reactions of tetracaine in the southeast of Iran (*n* = 24 patients, *n* = 31 eyes).


**Variable**	***N*** ** (%)**
**Gender **	
Male	22 (91.7)
Female	2 (8.3)
**Occupation**	
Welder	14 (58.3)
Housewife	2 (8.3)
Construction worker or facility worker	6 (25.0)
Farmer	1 (4.2)
Driver	1 (4.2)
**Place of obtaining tetracaine**	
Pharmacy	24 (100.0)
**Route of obtaining tetracaine**	
Over the counter (OTC)	22 (91.7)
Prescription by general practitioner (GP)	2 (8.3)
**Source of tetracaine introduction**	
Family members	1 (4.2)
Co-worker	21 (87.5)
General practitioner	2 (8.3)
**Previous history of tetracaine use**	
Yes	12 (50.0)
No	12 (50.0)
**Reason for drug use (initial injury)**	
Ultraviolet keratitis (UV keratopathy)	14 (45.3)
Ocular foreign body	7 (22.6)
Dry eye symptoms	3 (9.6)
Thermal burns	2 (6.5)
Ocular foreign body + Ultraviolet keratitis	1 (3.2)
Glue in the eyes	1 (3.2)
Thermal and chemical burns	1 (3.2)
Bell's palsy	1 (3.2)
Corneal abrasion	1 (3.2)
**Clinical signs at the first examination**	
CED (Corneal epithelial defect)	28 (90.3)
Diffuse or patchy corneal infiltration	16 (51.6)
Corneal ring infiltration	10 (32.3)
Descemet folding	10 (32.3)
Corneal edema	7 (22.6)
PEE (Punctate epithelial erosion)	6 (19.4)
Corneal haze	5 (16.1)
Keratic precipitate	5 (16.1)
Conjunctival necrosis	2 (6.5)
Corneal clouding	1 (3.2)
Hypopyon	1 (3.2)
**Gender **	
**Patient's main complaints ** ***n*** ** (%)**	
Pain	29 (93.5)
Redness	7 (22.6)
Reduced vision	24 (77.4)
Foreign body sensation	2 (6.5)
Photophobia	9 (29.0)
Lid edema	1 (3.2)
Irritation	2 (6.5)
**Symptoms in first interview ** ***n*** ** (%)**	
Pain	29 (93.5)
Redness	29 (93.5)
Reduced vision	30 (96.8)
Foreign body sensation	2 (6.5)
Photophobia	30 (96.8)
Lid edema	1 (3.2)
Tearing	10 (32.3)


**Table 2:** Some characteristics of drug abuse and treatment of the patients who were referred to eye emergency department for adverse drug reactions of tetracaine in southeast of Iran (*n* = 24 patients).


**Variable**	Mean ± SD (95% CI*)	**Range**
Hospitalization days	6.5 ± 0.99 (4.5–8.5)	0–17
Time from starting the topical tetracaine to the manifestation of adverse events (day)	9.03 ± 1.7 (5.62–12.44)	1–35
Number of drops used per day	20.2 ± 2.9 (14.3–26.1)	2–60
Interval of drops (hr)	1.2 ± 0.2 (0.85–1.51)	0.25–3.5
Time from primary injury to the first visit (days)	10.0 ± 1.72 (6.4–13.5)	2–36
Number of drug bottles used	5.7 ± 1.44 (2.8–8.6)	1–30

*95% Confidence Intervals		

**Table 3:** Vision on admission, on discharge, and final visual acuity (VA) of the patients who were referred to eye emergency department for adverse drug reactions of tetracaine in southeast of Iran (*N* = 31 eyes).


**Visual acuity**	**Eyes on admission ** ***n*** ** (%)**	**Eyes on discharge ** ***n*** ** (%)**	**Final exam ** ***n*** ** (%)**
HM*	4 (12.9)	1 (3.2)	1 (3.2)
1 m	3 (9.7)	–	–
2 m	3 (9.7)	1 (3.2)	–
3 m	4 (12.9)	2 (6.4)	–
0.1	8 (25.8)	8 (26.0)	–
0.2	4 (12.9)	3 (9.7)	1 (3.2)
0.3	–	4 (13.0)	–
0.5	1 (3.2)	3 (9.7)	3 (9.7)
0.6	4 (12.9)	2 (6.4)	1 (3.2)
0.7	–	–	5 (16.2)
0.8	–	1 (3.2)	7 (22.6)
0.9	–	–	2 (6.4)
1.0 (10/10)	–	–	11 (35.5)
Unknown	–	6 (19.3)	–
**Summary and mean of vision**			
**Visual acuity**	**No. on admission (%)**	**No. on discharge (%)**	**No. on final exam (%)**
HM–CF* ( < 1/10)	14	4	1
0.1–0.3	12	15	1
0.4–0.6	5	9	4
0.7–1	0	1	25

${}^{*}$ Hand Motion-Count Finger m, meters			

**Figure 1:**
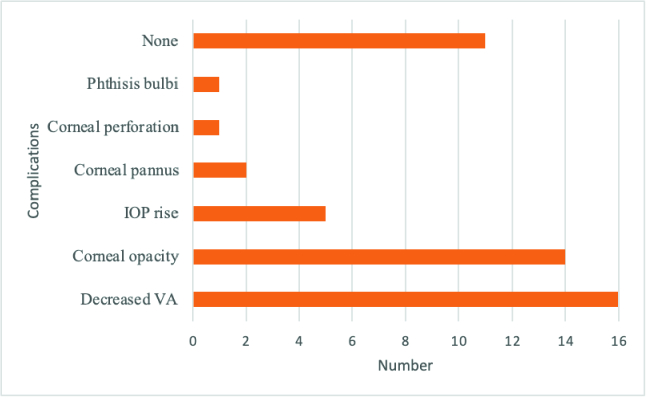
Final complications of adverse drug reactions of tetracaine of the patients who were referred to the eye emergency department for adverse drug reactions of tetracaine in southeast of Iran (*n* = 31 eyes). IOP, intraocular pressure; VA, visual acuity.

**Figure 2:**
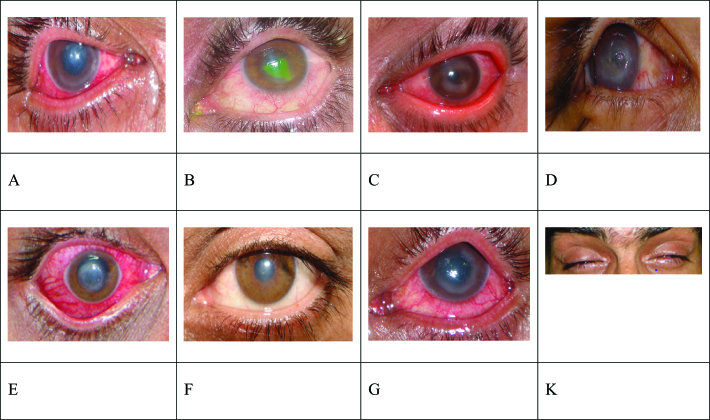
Slit photos in some patients. (A) Ring infiltration and diffuse patchy stromal infiltration; (B) Ring infiltration, diffuse patchy stromal infiltration, and PED (persistent epithelial defect); (C) Ring infiltration; (D) Seventh cranial nerve palsy, PED, diffuse stromal infiltration, and ring infiltration; (E) Central dense stromal infiltration; (F) Corneal scar and stromal haze following treatment of stromal infiltration; (G) Ring infiltration and stromal haziness; (K) Severe photophobia.

The final persistent complications were decreased visual acuity (in 51.6% of the cases) and corneal opacity (in 45.2% of the cases). There were no complications at the end in only 35.5% of the patients [Figure 1]. Of note, in this study, ocular conditions improved in all cases, except for one eye which belonged to a male patient with bilateral anesthetic toxicity that became phthisic following corneal perforation. This patient continued using the offending agent despite all recommendations to stop it.

##  DISCUSSION

We found some serious ADR related to using ophthalmic anesthetics. Misuse of ophthalmic anesthetics can cause severe ocular complications, leading to permanent effects such as corneal scars, vision loss, and even loss of the globe. In the current study, the most common clinical findings were PED, diffuse or patchy corneal infiltrations, Descemet folding, or corneal ring infiltrations. Moreover, the most persistent morbidities were reduced vision, corneal opacity, and elevated intraocular pressure. In a previous study, Rosenwasser et al reported six individuals with topical anesthetic toxicity suffering from PEDs, corneal stromal ring infiltrates, and disproportional pain. Penetrating keratoplasty was done for the treatment of corneal perforation in two cases. Eventually, two eyes were enucleated. Of the six patients, five cases were mistakenly diagnosed and treated as *Acanthamoeba keratitis.* ^[1]^ A case report in Iran reported complications due to misuse of tetracaine in a 42-year-old man. Bilateral Mooren-like ulcers, low vision, severe pain bilaterally, bilateral epithelial defects, and stromal ring infiltration were observed. After treatment, low visual acuity, bilateral epithelial defects, and stromal ring infiltration remained. This study reported that corneal haze and hypopyon were considerable.^[8]^ Persistent corneal epithelial defect, a ring infiltrate, epithelial defect with diffuse stromal edema, Punctate Epithelial Erosion (PEE), low vision, and dense stromal infiltration were observed after misusing tetracaine in a 32-year-old man.^[9]^

It was demonstrated that the most common ocular problems that led to the abuse of topical anesthetics by patients were ultraviolet keratitis and ocular surface foreign bodies. Other cases also experienced unusual dry eye symptoms, Bell's palsy, ocular surface exposure to glue, corneal abrasion, and thermal burns. In a case series conducted by Yagci et al, the initial injuries in patients included ultraviolet keratitis, ocular foreign bodies, and chemical injury.^[10]^ Goldich et al also reported a man with floppy eyelid syndrome and topical anesthetic abuse. They believed that these cases with unusual causes may need psychiatric consultation due to their problems.^[11]^ Katsimpris et al reported the results of misuse of tetracaine in a 34-year-old man, which included intense hyperemia of the conjunctiva, corneal epithelial defect, complete ring infiltrate, and elevated intraocular pressure. [12]

In our study, the mean duration of tetracaine abuse was 9.03 days (ranging from only two drops up to 30 bottles of the drug) before seeking the appropriate therapy. In a report by Erdem et al, the mean duration of anesthetic abuse was around 15 days.^[13]^ Furthermore, the mean duration of abuse in Tok et al's study was about 18 days.^[14]^ This prolonged use of medication can cause a significant delay in both the diagnosis and application of the appropriate treatment. It can also cause an occurrence of a vicious cycle that in turn leads to overuse of medicine and aggravation of serious complications, especially in those patients who deny ophthalmic drug misuse. In many patients, drug abuse may be continued even after hospitalization, where ocular manifestations may be misdiagnosed as infectious or acanthamoeba keratitis.^[15]^ Yagci et al reported a series of 19 cases, 12 of whom had continued abuse of drops during their hospitalization time.^[10]^ All of our patients disclosed the misuse of ophthalmic drops in their first examination. Since topical anesthetics have both dose- and time-dependent cytotoxic effects on corneal epithelial cells,^[16]^ it is important to discontinue them to reduce morbidities. Therefore, it is necessary in cases with clinical pictures suggesting toxicity or presence of unusual presentations to be strongly suspicious of the possibility of misuse of ophthalmic anesthetics. Despite the recommendations to stop applying the topical anesthetics, one of our patients continued using drops even after several weeks from the diagnosis. Eventually, corneal perforation happened in one eye. The patient refused to be admitted for tectonic keratoplasty; as a consequence, phthisis bulbi and permanent loss of vision happened.

Some of the patients showed corneal adverse effects even with minimal use of tetracaine eye drops for a short-term. In one patient, bilateral diffuse corneal haziness and corneal epithelial defect with severely reduced vision occurred after using only two drops of 0.5% tetracaine ophthalmic preparation. However, some references recommended the short-term use of topical anesthetics for pain relief after performing surgical procedures such as photorefractive keratectomy or treating pain resulting from corneal abrasions.^[17]^ Correspondingly, this is alarming for the magnitude of problems that may happen even with short-term use and a low-dosage of such drugs, suggesting that these drugs are not necessarily safe. Chen et al, in their research, reported a woman with toxic keratopathy following an application of a low concentration (0.05%) of oxyuricide. After that, they advised that topical ocular anesthetics never be prescribed.^[18]^

Since there is no consensus over an effective strategy to treat ocular lesions, especially corneal lesions resulting from abuse or misuse of the offending medications, the most effective strategy seems to be taking preventative measures to minimize abuse of these agents. Accordingly, prevention of easy access to medication can be helpful. In the present study, the protocol introduced for the treatment of the patients resulted in a significant improvement of vision from admission to discharge time and eventually up to the last post-treatment examination.

In this study, the main signs of ADR from misuse of an anesthetic were corneal epithelial defect, patchy or diffuse corneal stromal infiltration, and stromal ring infiltrations. In a study conducted by Yagci et al, ring-shaped infiltration and stromal infiltration were reported as the main findings in this regard.^[10]^ The main finding by Erdem et al was ring infiltration.^[13]^ It is necessary to maintain a high awareness for anesthetic abuse among patients diagnosed with diffuse and longstanding epithelial, as well as stromal and endothelial corneal lesions because these presentations are usually misdiagnosed as infectious and acanthamoeba keratitis.^[2]^

Most of our patients in this study obtained the drugs with no prescription, illegally, from pharmacies, but a few patients received prescriptions by general practitioners. In developing countries, many agents even with proven toxic effects, are sold in pharmacies. Erdem et al, in a case series study performed in Turkey, reported one patient that obtained the drug with a prescription, and for others, it was available with no prescription.^[13]^ So, in order to address this problem, the healthcare system authorities need to regulate and apply limitations to the distribution and access of these types of drugs.

One limitation of this study was loss of regular follow-up of some patients, and consequently the loss of data. The other limitation was recall bias, as some patients could not remember some variables like the exact time of starting the treatment.

In summary, misusing topical ophthalmic anesthetics leads to severe, permanent, and sight-threatening debilitating effects, including reduced visual acuity, corneal opacity, corneal perforation, elevated intraocular pressure, and loss of the globe. The healthcare system should control and restrict the OTC access to toxic medications such as tetracaine eye drops. Although tetracaine eye drop is categorized as a prescription medication in Iran, pharmacies provide it, illegally, over-the-counter to the patients. In addition, some general practitioners are not aware of the dangers of this medication; so, it is necessary to restrict the access of the medicine without prescriptions from ophthalmologists, and it is recommended that the medicine become a true non-OTC and its availability in pharmacies and drug stores be legally regulated. General practitioners and ophthalmologists should pay more attention to the adverse effects of anesthetics and consider the possible risks of prescribing these medications. It is also necessary to educate the public especially those at a higher risk, like welders who represent the majority of users.

##  Financial Support and Sponsorship

None.

##  Conflicts of Interest

None of the authors have any conflicts of interest related to this study**.**
